# Supplementary figures and images for: The effect of memantine, an antagonist of the NMDA glutamate receptor, in in vitro and in vivo infections by Trypanosoma cruzi
Source: PLoS Negl Trop Dis. 2019 Sep 19;13(9):e0007226. doi: 10.1371/journal.pntd.0007226 (PMC6752752; doi:10.1371/journal.pntd.0007226)

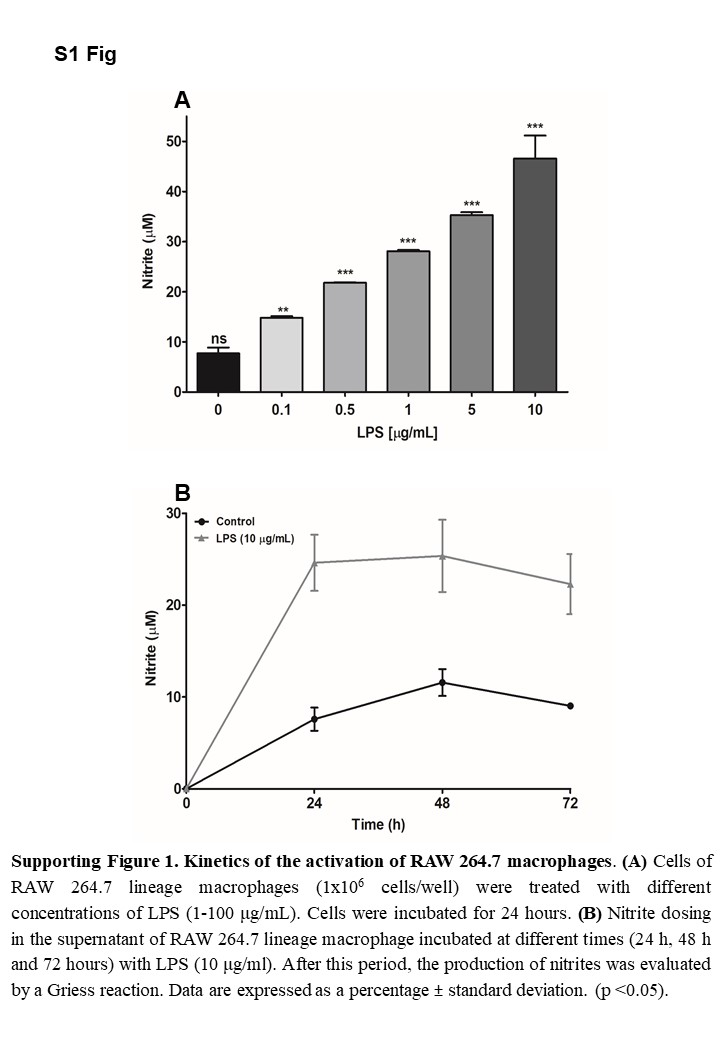

Supplement: S1 Fig — (A) Cells of RAW 264.7 lineage macrophages (1x10^6^ cells/well) were treated with different concentrations of LPS (1–100 μg/mL). Cells were incubated for 24 hours. (B) Nitrite dosing in the supernatant of RAW 264.7 lineage macrophages incubated at different times (24, 48 and 72 hours) with LPS (10 μg/ml). After this period, the production of nitrites was evaluated by a Griess reaction. Data are expressed as a percentage ± standard deviation. (p <0.05). (JPG) [file pntd.0007226.s001.JPG]

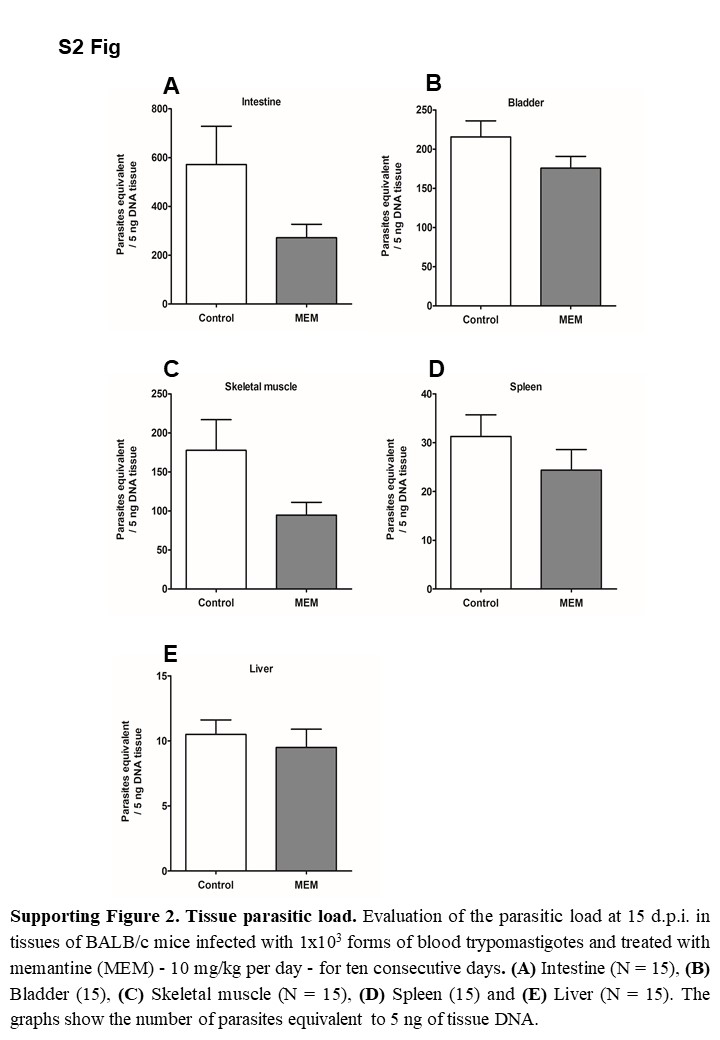

Supplement: S2 Fig — Evaluation of the parasitic load at 15 d.p.i. in tissues of BALB/c mice infected with 1x10^3^ forms of blood trypomastigotes and treated with memantine (MEM) - 10 mg/kg per day—for ten consecutive days. (A) Intestine, (B) Bladder, (C) Skeletal muscle, (D) Spleen and (E) Liver. The experiments were repeated four times with 10 animals/group. The graphs show the number of parasites equivalent to 5 ng of tissue DNA. (JPG) [file pntd.0007226.s002.JPG]
